# Searching for optimal machine learning model to classify mild cognitive impairment (MCI) subtypes using multimodal MRI data

**DOI:** 10.1038/s41598-022-08231-y

**Published:** 2022-03-11

**Authors:** Tatsuya Jitsuishi, Atsushi Yamaguchi

**Affiliations:** Department of Functional Anatomy, Graduate School of Medicine, Chiba University, 1-8-1 Inohana, Chuo-ku, Chiba, 260-8670 Japan

**Keywords:** Neuroscience, Systems biology, Medical research, Neurology

## Abstract

The intervention at the stage of mild cognitive impairment (MCI) is promising for preventing Alzheimer’s disease (AD). This study aims to search for the optimal machine learning (ML) model to classify early and late MCI (EMCI and LMCI) subtypes using multimodal MRI data. First, the tract-based spatial statistics (TBSS) analyses showed LMCI-related white matter changes in the Corpus Callosum. The ROI-based tractography addressed the connected cortical areas by affected callosal fibers. We then prepared two feature subsets for ML by measuring resting-state functional connectivity (TBSS-RSFC method) and graph theory metrics (TBSS-Graph method) in these cortical areas, respectively. We also prepared feature subsets of diffusion parameters in the regions of LMCI-related white matter alterations detected by TBSS analyses. Using these feature subsets, we trained and tested multiple ML models for EMCI/LMCI classification with cross-validation. Our results showed the ensemble ML model (AdaBoost) with feature subset of diffusion parameters achieved better performance of mean accuracy 70%. The useful brain regions for classification were those, including frontal, parietal lobe, Corpus Callosum, cingulate regions, insula, and thalamus regions. Our findings indicated the optimal ML model using diffusion parameters might be effective to distinguish LMCI from EMCI subjects at the prodromal stage of AD.

## Introduction

Alzheimer's disease (AD) is the most common cause of dementia developing over a period of years, characterized by cognitive and behavioral problems (NIH,https://www.ninds.nih.gov/). The mild cognitive impairment (MCI) is considered as a transitional stage between aging and AD^1^. There is no definitive cure available to date when subject is once diagnosed AD, which is in the later disease stage. The early detection and therapeutic intervention at the preclinical or prodromal stage is promising to prevent dementia. The pathophysiological process of AD reportedly starts two decades or more before symptoms. One-third of MCI develop AD within five years’ follow-up. Therefore, this preclinical or prodromal phase, especially at the MCI stage, provides an opportunity for preventive intervention^2,3^. Alzheimer’s disease neuroimaging initiative (ADNI) is the multisite observational study of normal aging, MCI, and AD. MCI subjects are sub-classed in two subtypes, early MCI (EMCI) and late MCI (LMCI) in ADNI, based on the WMS-R Logical Memory II Story A score. The EMCI is considered to reflect those at the earlier point in the clinical spectrum, while LMCI is at the later point to progress to AD^4–6^. Since EMCI and LMCI subtypes were classified by the severity of amnestic impairment through a single memory score, it could account for low specificity and even misclassifications. To find potentially high-sensitive biomarkers that change with disease progression might assist the more precise disease staging, which can reduce the number of AD patients through early intervention. Especially, the stage of EMCI might be optimal for disease-modification interventions. Thus, there is an increasing amount of attention to identify the subtle alterations among MCI subjects^4–10^.

The integrity of white matter microstructure is commonly assessed with fractional anisotropy (FA) and mean diffusivity (MD) owing to anisotropic proton diffusion. The directional dependence of proton diffusion is quantified as FA, while the magnitude of diffusivity is quantified as MD. The reduced FA and elevated MD would reflect the neuronal loss and disruption of myelin sheaths in degenerative brains^11–13^. The tract-based spatial statistics (TBSS) was developed as a voxel-wise analysis to improve the sensitivity, objectivity, and interpretability of multi-subject diffusion imaging data by the statistics with FA skeleton^14^. On the other hand, the functional MRI is based on the blood oxygenation level-dependent (BOLD) signals to assess the neural activity in different parts of the brain^15,16^. The functional connectivity of default mode network (DMN) was selectively altered in AD patients as well as MCI subjects by resting-state functional MRI (rs-fMRI) analyses^17–19^. Several graph-theoretical parameters (e.g. global or local efficiency, small-worldness) have been used to measure characteristics of functional brain networks. Although conclusions are inconsistent, AD patients showed alterations in the functional segregation, hub connectivity, modular integrity, and/or the small-world network^20–22^.

Several studies with machine learning (ML) approach have applied single- or multi-modal neuroimaging data for classification of MCI subtypes. Gray et al. used the information of FDG-PET (^18^F-fluorodeoxyglucose-positron emission tomography)^23^. Nozadi et al. also used PET images for classification, comparing FDG and Amyloid **(**AV-45) PET biomarkers^24^. Shi and Liu extracted features from rs-fMRI signals^25^, and Sheng et al. processed thousands of brain network features by graph theory for classification^26^. Wee et al. have indicated the multi-modal neuroimaging approach with structural and functional connectivity analyses significantly improves the identification accuracy of MCI^27^. Goryawala et al. combined MRI volumetric measures with neuropsychological scores to classify MCI subtypes^28^. In general, the white matter damage, measured by diffusion MRI, is considered to precede grey matter atrophy in AD patients^29,30^. However, the number of ML models using diffusion MRI is limited to classify MCI subtypes.

In the present study, we hypothesized the subtle brain alterations could be detected earlier in the white matter microstructures of MCI subjects, which prompted us to search for optimal diffusion MRI-based ML models for EMCI/LMCI classification. We first investigated the alterations in the white matter integrity by TBSS in MCI subjects. Next, we addressed the connected cortical areas by ROI-based tractography. Then to obtain features for classification, we analyzed the resting-state functional connectivity (RSFC) and graph-theoretical metrics by rs-fMRI. We also prepared two feature subsets of diffusion parameters (FA, MD) in the regions of LMCI-related white matter changes detected by TBSS. Using these four feature subsets, we employed multiple ML models for EMCI/LMCI classification and assessed the performance with cross-validation.

## Material and methods

### Ethical statement

All individual imaging data, shared publicly with general scientific community, was obtained from Alzheimer’s Disease Neuroimaging Initiative (ADNI) based on DATA USE AGREEMENT (http://adni.loni.usc.edu/). All methods and protocols were approved by the Research Ethics Committee of Chiba University School of Medicine.

All the methods were performed in accordance with relevant guidelines and regulations.

### ADNI participants

Data used in the present article was obtained from the ADNI database (adni.loni.usc.edu). The ADNI was launched in 2003 as a public–private partnership, led by Principal Investigator Michael W. Weiner, MD. For up-to-date information, see www.adni-info.org. ADNI-3 began in 2016 and includes scientists at 59 research centers in the United States and Canada. To ensure sufficient statistical power to assess differences in data collected with different protocols in each scanning site, we used the available data only in ADNI-3 at the time of download. This study reflects the data available on December 2020. In ADNI, MCI subject is diagnosed on the criteria; (1) subjective memory concern reported by the participant, study partner, or clinician; (2) abnormal memory function documented by scoring within the education adjusted ranges on the Logical Memory II subscale (Delayed Paragraph Recall, Paragraph A only) from the Wechsler Memory Scale-Revised (the maximum score of 25); (3) Mini-Mental State Examination (MMSE) score between 24 and 30; (4) global Clinical Dementia Rating (CDR) score of 0.5, with a Memory Box score of at least 0.5; and (5) general cognition and functional performance sufficiently preserved such that a diagnosis of AD could not be made. Participants used in the present study were 34 and 32 individuals diagnosed with early MCI (EMCI) and late MCI (LMCI) respectively, based on the WMS-R Logical Memory II Story A score. The EMCI subjects were recruited with memory function approximately 1.0 SD below, while those of LMCI were approximately 1.5 SD below expected education adjusted norms^2,4,5,7^. The specific cutoff scores were as follows (a maximum score of 25): EMCI was diagnosed for a score of 9–11 for 16 or more years of education; a score of 5–9 for 8–15 years of education; or a score of 3–6 for 0–7 years of education. LMCI was diagnosed for a score of 8 for 16 or more years of education; a score of 4 for 8–15 years of education; or a score of 2 for 0–7 years of education. Demographic and neuropsychological information in this study were shown in Table 1 and Supplementary Figs. S1, S2.

**Table 1:** Demographic and neuropsychological Information from ADNI-3 dataset.

	Gender (M/F)	Age (mean ± SD)	MMSE (mean ± SD)	MoCA (mean ± SD)	ADAS-Cog (mean ± SD)
EMCI (n = 34)	24/10	75.2 ± 7.1 [62–91]	27.85 ± 2.95 [18–30]	22.97 ± 3.97 [14–29]	13.79 ± 10.34 [0.33–48]
LMCI (n = 32)	16/16	75.8 ± 6.5 [61–85]	24.72 ± 6.34 [9–30]	21.29 ± 6.86 [2–29]	19.24 ± 13.39 [1.6–54.6]

*MMSE* Mini-mental State Examination, *MoCA* Montreal Cognitive Assessment, *ADAS-Cog* AD Assesment Scale-Cognitive Scale, *SD* standard deviation [min–max].

### MRI acquisition protocols in ADNI

ADNI-3 imaging is done exclusively on 3 T scanners. The MRI acquisition of ADNI-3 consists of Participant Scan (3 Plane Localizer, Accelerated Sagittal MPRAGE, Sagittal 3D FLAIR, Axial T2 STAR, Axial 3D PASL, Axial DTI, Field Mapping, Axial rs-fMRI, HighResHippocampus) and Phantom Scan (3 Plane Localizer, QC Phantom MPRAGE). The scanning protocols of T1-weighted MRI (voxel size = 1 mm^3^), diffusion-weighted image (DWI) (voxel size = 2 mm^3^), functional MRI are described in detail on the ADNI website (http://adni.loni.usc.edu/methods/mri-tool/mri-analysis/). ADNI-3 utilized diffusion MRI protocols for 3 T Siemens, Philips, and GE scanners, using 2.0 mm isotropic voxels with b = 0 and 1000 s/mm^2^ weighted volumes. The DICOM images, acquired from ADNI-3 database, were converted to NIFTI format with the *dcm2nii* part of MRIcroGL (https://www.nitrc.org/projects/dcm2nii/).

### Diffusion MRI preprocessing

Diffusion MRI data were preprocessed using MRtrix3.0^31^, FSL 6.0 (www.fsl.fmrib.ox.ac.uk)^32^, and advanced normalization tools (ANTs). We conducted the preprocessing process based on the recommendations by Maximov et al.^33^. The following steps were conducted: (1) noise correction using Marchenko-Pastur principal component analysis (MPPCA) ('dwidenoise'; MRtrix3.0 command), (2) correction for Gibbs ringing artifacts ('mrdegibbs'; MRtrix3.0 command), (3) motion correction, eddy current, and susceptibility distortion correction ('dwifslpreproc'; MRtrix3.0 command), (4) bias field correction calculated by advanced normalization tools (ANTs), (5) DTIFIT in FSL fits a diffusion tensor model at each voxel on the pre-processed diffusion image.

### Diffusion MRI tractography

Deterministic fiber tracking was conducted as previously described^34,35^. Briefly, the reconstruction of tractography was performed by ROI (region of interest)-based approach with DSI Studio (http://dsi-studio.labsolver.org). After fiber tracts were generated by whole-brain seeding, the tracts running through ROIs were selected for analysis. The parameters for fiber tracking included a step size of 0.2 mm, a minimum and maximum fiber length of 20 mm and 800 mm respectively, and a turning angle threshold of 60°. This progression was repeated until the quantitative anisotropy (QA) of the fiber orientation dropped below the default threshold, until fiber tract continuity no longer met the progression criteria, or until tracking reached to 10,000,000 seeds^34–36^.

The HCP-MMP1.0 was used for the parcellation of cerebrum, which is a surface-based coordinate system (“greyordinates”) created in the CIFTI format^37^. In this study, the built-in HCP MMP1.0 atlas of DSI Studio was used to convert all 180 areas from a surface-based coordinate system to volumetric coordinates.

The quantitative tractography analysis was conducted, in which the ‘connectivity matrix’ function in DSI Studio was used to generate matrices representing the number of fibers ending in regions of a per-subject aligned HCP MMP1.0 atlas. After the bilateral connectivity matrices were generated, the number of streamlines corresponding to each connection was divided by the total number of each tract^34,35^.

### HCP1065 template

The HCP 1065 template was constructed from a total of 1065 subjects' diffusion MRI data from the Human Connectome Project (2017 Q4, 1200-subject release). The HCP1065 data are shared under the WU-Minn HCP open access data use term. The HCP1065 registration is based on the nonlinear ICBM152 2009a space. A multishell diffusion scheme was used, and the b-values were 1000, 2000, 3000 s/mm^2^. The number of diffusion sampling directions was 90, 90, and 90, respectively. The in-plane resolution was 1.25 mm, with the slice thickness was 1.25 mm. The diffusion data were reconstructed in the MNI space using q-space diffeomorphic reconstruction to obtain the spin distribution function^36^. A diffusion sampling length ratio of 1.7 was used, and the output resolution was 1 mm. The analysis was conducted using DSI Studio (http://dsi-studio.labsolver.org).

### Tract-based spatial statistics (TBSS)

The preprocessed diffusion MRI imaging data from ADNI-3 were further processed with the DSI Studio (http://dsi-studio.labsolver.org). The diffusion data were reconstructed in the MNI space using q-space diffeomorphic reconstruction (QSDR), an extension of the generalized q-sampling imaging (GQI), to obtain the spin distribution function (SDF)^36^. GQI obtain the SDF from the shell sampling scheme used in q-ball imaging (QBI), which is more sensitive to intravoxel orientational heterogeneity than classical diffusion tensor imaging (DTI) algorithm. Generalized fractional anisotropy (gFA) is considered as the QBI analog of DTI-derived FA^38^, which is the most widely used QBI measure. Since Corbo et al. (2014) showed the advantage of gFA-based TBSS compared to FA-based TBSS, we conducted gFA-based TBSS using gFA instead of FA as described previously^39^. In this study, ‘gFA’ means generalized FA, while ‘FA’ means DTI-FA.

After obtaining the gFA or MD (mean diffusivity) image from reconstructed diffusion data by DSI studio, we conducted the voxel-wise statistical analysis of gFA or MD data using TBSS (Tract-Based Spatial Statistics) of FSL, respectively^14,32^. TBSS projects all subjects' gFA or MD data onto the mean gFA or MD tract skeleton respectively, before applying voxelwise cross-subject statistics. TBSS aims to improve the sensitivity, objectivity, and interpretability of analysis of multi-subject diffusion imaging studies (https://fsl.fmrib.ox.ac.uk). For all TBSS analyses, *p* < 0.05 was considered significant. Since the null distribution is not known, nonparametric permutation tests were used for thresholding on statistic maps to detect differences in FA between EMCI and LMCI subjects. Threshold-free cluster enhancement (TFCE) was applied to find significant clusters of voxels (*p* < 0.05) and correct multiple comparisons for family-wise error (FWE).

### Resting-state functional MRI (rs-fMRI)

Functional connectivity (FC) was analyzed with CONN-fMRI toolbox for the Statistical Parametric Mapping (SPM12), which is a MATLAB-based cross-platform software (http://www.conn-toolbox.org). Briefly, the resting-state data, band-pass filtered (0.008–0.09 Hz), were processed by CONN, including slice-timing correction, realignment, individual structural–functional image co-registration, MNI template normalization, and spatial smoothing. White matter, CSF (cerebrospinal fluid), and physiological noise source reduction were taken as confounders with the implemented CompCor strategy^40^.

The ROI-to-ROI fMRI analysis basically computes the temporal correlation of BOLD activity between distinct regions from a given area to all other areas using a General Linear Model (GLM) approach. For the segmentation of cortical areas, DSI studio-built in HCP MMP1.0 atlas (http://dsi-studio.labsolver.org) was incorporated to CONN for FC analyses. All FC measures were available in CONN for each subject and each condition (first-level analyses). Subject-specific contrast images reflecting standardized correlation coefficients were obtained for further analyses. The correlation coefficient (*r*) was converted to the normally distributed variable (*z*) by Fisher's *z*-transformation.

### Graph measures (ROI-level)

With the graph theory analysis in CONN-toolbox (https://web.conn-toolbox.org/fmri-methods/connectivity-measures/graphs-roi-level), we explored resting-state functional connectivity (RSFC) between brain areas by the ROI-to-ROI approach. All ROI-level graph measures are based on nondirectional graphs with nodes (ROIs) and edges (suprathreshold connections). For each subject, a graph adjacency matrix *A* is computed by thresholding the associated ROI-to-ROI Correlation (RRC) matrix *r* by an absolute or relative threshold. Then, based on the resulting graphs, a number of measures can be computed addressing topological properties of each ROI within the graph as well as of the entire network of ROIs^41,42^.

### Machine learning (ML)–based classifications

We utilized the scikit-learn (https://scikit-learn.org/), a library for machine learning (ML) in Python 3, to conduct multiple ML classification algorithms^43^. Based on the selected feature subsets, ML models were adopted by using several classifiers, including support vector machine (SVM), K-nearest neighbor (KNN), logistic regression (LR), random forest (RF), gradient boosting classifier (GBC), and Adaptive boosting (AdaBoost)^27,44,45^. SVM, a supervised learning method, searches for an optimal separating hyperplane between classes, which maximizes the margin. LR is the statistical technique used to predict the relationship between the dependent and the independent variable, where the dependent variable is binary in nature. K-nearest neighbors (KNN), a type of supervised learning algorithm, tries to predict the correct class for the test data by calculating the distance between the test data and all the training points. RF, GBC, and AdaBoost are ensemble ML algorithms, based on the idea of creating a highly accurate prediction rule by boosting or bagging many relatively weak and inaccurate rule to improve generalizability/robustness over a single estimator^44,45^.

Ten-fold cross-validation (CV) was used for the evaluation of each ML model^27^. We used the stratified cross-validator ‘StratifiedKFold (n_splits = 10, random_state = 0)’ of scikit-learn tool (https://scikit-learn.org/) in all the evaluations, which enabled us to compare the classification performance based on the same conditions. The test portion was hold out exclusively for testing (evaluation). Briefly the data were split into ‘training’ and ‘test’ sets. Models were trained using only the ‘training’ set, and model performance was assessed using the only ‘test’ set. The classification performance of different classifiers was evaluated using accuracy (ACC), precision, recall, F1 score, which were calculated based on the confusion matrix of classification results. The area under the receiver operating characteristic curve (AUC(ROC)) was calculated using ‘roc_ auc’ in the scikit-learn tool (https://scikit-learn.org/). We took the mean of each metric to evaluate classification performance. The definitions of ACC, precision, recall, and F1 score are given as follows: ACC = (TP + TN)/(TP + TN + FP + FN), precision = TP/(TP + FP), Recall = TP/(TP + FN), F1 score = 2 * TP/(2 * TP + FP + FN), where TP, TN, FP, and FN represent the numbers of true positive, true negative, false positive, and false negative, respectively^27,45^.

### Feature extraction

Total four feature subsets were prepared for ML-based classifiers per subject. Each subject’s feature subset contained 12–36 feature columns and the last column of class labels (i.e. EMCI; label 0, LMCI; label 1). *FA-based TBSS, MD-based TBSS method*; We measured FA and MD values in the cortical areas of LMCI-related white matter changes, detected by gFA-based TBSS (a feature vector of 14 elements; 14 = 7 regions x (mean FA + MD)) and MD-based TBSS (a feature vector of 24 elements; 24 = 12 regions x (mean FA + MD)), respectively. *TBSS-RSFC, TBSS-Graph method*; We first addressed the cortical areas that were connected by affected callosum fibers, and then we conducted rs-fMRI analyses to calculate the resting-state functional connectivity (RSFC) and graph-theoretical metrics in those cortical areas, respectively. Using ROI-to-ROI binary correlation matrix (360-by-360 matrix based on HCP-MMP1.0 atlas) in each subject, we obtained the functional correlation coefficient (*r*) between connected cortical areas, which was transformed to *z* value with Fisher *r*-to-*z* transformation (i.e. TBSS-RSFC method). The TBSS-RSFC method resulted in a feature vector with 12 elements/subject (12 = correlation coefficient (*z*) × 12 connected areas). We also measured the 2 graph-theory metrics of representative functional segregation (i.e. clustering coefficient, local efficiency) in the cortical areas by applying graph theory on rs-fMRI analyses (i.e. TBSS-Graph method) with CONN-fMRI toolbox for SPM12. The TBSS-Graph method resulted in a feature vector with 36 elements/subject (36 = 2 graph-theory metrics × 18 HCP-MMP1.0 areas).

## Results

### The LMCI-related white matter (WM) alterations by gFA-based TBSS

**Figure 1:**
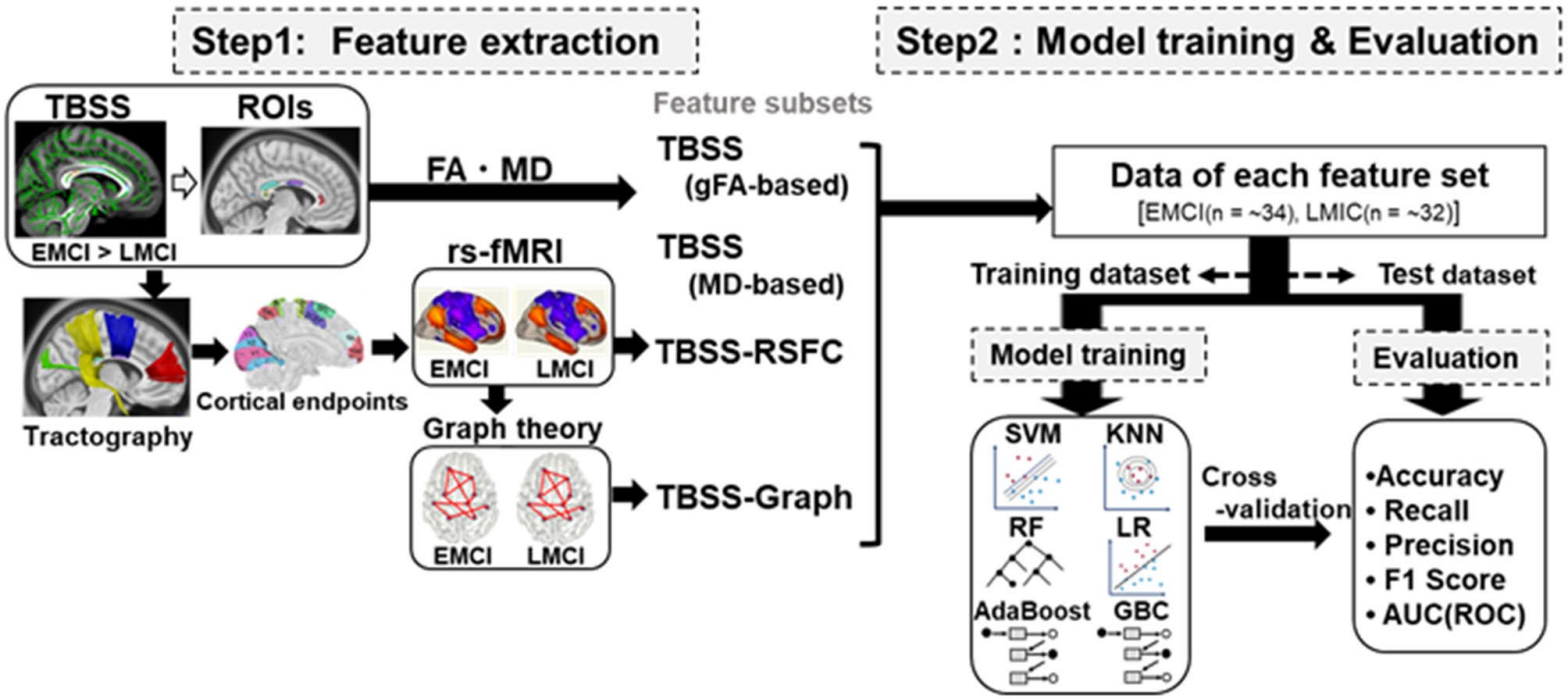
Flow chart representing Machine learning (ML) approach for EMCI/LMCI classification. The flow chart represents the framework of machine learning (ML) algorithm for EMCI/LMCI classification. Step1 consists of feature extraction by multi-modal methods, including TBSS, tractography, RSFC, and graph theory. Step 2 consists of ML models (SVM, KNN, LR, DTC, RF, GBC, AdaBoost) with tenfold cross-validation (CV). The dataset was divided into training and test dataset for tenfold CV, calculating the mean ‘accuracy (ACC)’, ‘recall’, ‘precision’, ‘F1 score’, and ‘AUC(ROC)’. *MCI* mild cognitive impairment, *EMCI* early MCI, *LMCI* late MCI, *FA* fractional anisotropy, *MD* mean diffusivity, *TBSS* Tract-based spatial statistics, *RSFC* resting-state functional connectivity, *CV* cross-validation, *ROI* range of interest, *ML* Machine learning, *KNN* k-nearest neighbor algorithm, *LR* Logistic Regression, *DTC* Decision Tree Classification, *RF* Random Forest, *SVM* support vector machine, *GBC* gradient boosting classifier, *AdaBoost* Adaptive Boosting, *ACC* accuracy, *ROC* Receiver operating characteristic, *AUC* Area under the curve.

**Figure 2:**
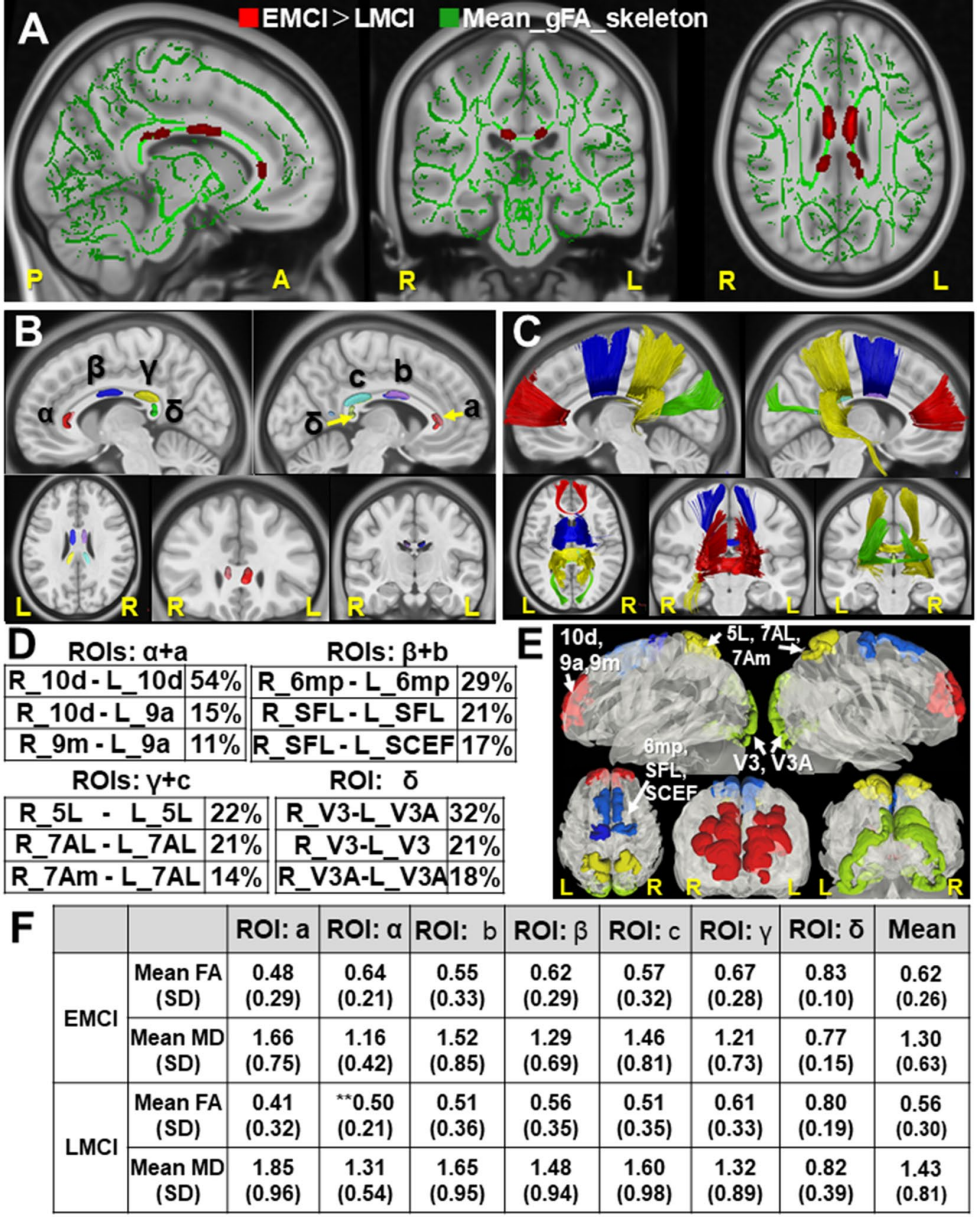
Sequential integration of TBSS and Tractography analyses. (**A**) The gFA (generalized fractional anisotropy)-based TBSS projects all subjects' gFA data onto a mean gFA tract skeleton before applying voxelwise cross-subject statistics (EMCI vs. LMCI). The registered average subjects’ gFA tract skeleton is represented in green, while LMCI-related white matter changes were represented in red color. The mean gFA tract skeleton was overlaid on the sagittal, coronal, and axial T1-weighted MRI image (ICBM average brain). Left; sagittal view of the left hemisphere, Middle; coronal section, Right; axial view. Significance level was *p* < 0.05 (EMCI vs. LMCI, Threshold Free Cluster Enhancement and Family-Wise Error corrected). (**B**) The ROIs for fiber tracking, identified as white matter alterations by gFA-based TBSS (EMCI vs. LMCI), were shown overlaid on the sagittal, axial, and coronal T1-weighted MRI image (ICBM average brain), respectively. The α, β, γ, and δ indicate the ROIs in the Corpus Callosum of the left hemisphere, while the a, b, and c indicate those in the right hemisphere. (**C**) Tractogram, using the altered white matter regions as ROIs, was shown overlaid on the sagittal, axial, and coronal T1-weighted MRI image (ICBM average brain), respectively. The streamlines passing through the ROIs (α, a), ROIs (β, b), ROIs (γ, c), and ROI (δ), were shown in red, blue, yellow, and green, respectively. (**D**) The tables show the top 3 of cortical areas (%, number of streamlines/total of each tract) identified by endpoint analyses, into which the callosal fibers project inter-hemispherically. (**E**) Cortical areas used for TBSS-RSFC and TBSS-Graph method, overlaid on the 3D glass brain (HCP1065). The regions in red are cortical areas in the frontal lobe (i.e. 10d, 9a, 9m), those in blue are in the precentral region (i.e. 6mp, SFL, SCEF), those in yellow are in the parietal lobe (i.e. 5L, 7AL, 7Am), and those in green are in the occipital lobe (i.e. V3, V3A). (**F**) The table shows mean diffusion parameters (mean FA, MD) in each ROI of LMCI-related white matter changes, which were sub-classed into the ROI of α, a, β, b, γ, c, and δ. **p < 0.05 (EMCI vs. LMCI, *t*-test). Statistical data are in Supplementary Fig. S3. *FA* fractional anisotropy, *MD* mean diffusivity, *TBSS* Tract-based spatial statistics, *ML* machine learning, *MCI* mild cognitive impairment, *EMCI* early MCI, *LMCI* late MCI, *RSFC* resting-state functional connectivity, *ROI* range of interest, *SD* standard deviation.

To search for the optimal ML model for EMCI/LMCI classification, we first used diffusion MRI dataset in ADNI database (as flowchart in Fig. 1). The gFA-based TBSS indicated the LMCI-related white matter (WM) changes in the Corpus Callosum (CC), the largest bundles of commissural fibers (Fig. 2A). The LMCI-related WM changes, shown in Fig. 2A, were sub-classed into the anterior ROIs (α, a), middle ROIs (β, b), and posterior ROIs (γ, c, and δ) in the CC (Fig. 2B). Then to address which cortical areas are possibly connected by the callosal fibers inter-hemispherically, we conducted fiber tracking using the areas of LMCI-related WM changes as ROIs in the template brain (HCP1065) (Fig. 2C). We then quantified the cortical areas in which each bundle of streamlines project by cortical endpoint analyses. The tables in Fig. 2D listed the top 3 of cortical areas connected by streamlines running through each pair of ROIs, which were represented overlaid on the template brain (Fig. 2E). The cortical endpoint analyses showed the streamlines, passing through the ROIs (α-a), connect the frontal superior and middle gyri (10d, p10p, 9p, 9a) in the frontal lobes (Fig. 2C,E). Those, passing through the ROIs (β, b), connected superior motor and precentral areas (SFL, SCEF, 6mp) (Fig. 2C,E). Those, passing through the ROIs (γ-c) and (δ), connect the cortical regions, including paracentral, postcentral cortices (3b, 5mv), precuneus (7Am), superior parietal lobes (5L, 7AL, 7PC), and occipital visual areas (V3, V3A), respectively (Fig. 2C,E). The table in Fig. 2F indicates the mean diffusion parameter (mean FA and MD) in each ROI of LMCI-related WM changes. Although individual variation exists between each region, reduced FA value was observed in ROI α in LMCI subjects, compared with EMCI subjects (*p* < 0.05, *t*-test). Statistical data of Fig. 2F is in Supplementary Fig.S3.

### Feature extraction by ROI-based and Graph theory-based RSFC

Then to extract features to classify EMCI/LMCI subjects by machine learning (ML), we conducted rs-fMRI analyses to calculate the resting-state functional connectivity (RSFC) and graph theory metrics in the above cortical areas (Fig. 2E), respectively. At first, using ROI-to-ROI binary correlation matrix in each subject, we obtained the functional correlation coefficient (*z*) between connected cortical areas (i.e. TBSS-RSFC method) (12 elements = 1 coefficient (z) × 12 connected areas in Fig. 2D). On the other hand, previous graph theory-based studies showed the functional segregation is impaired in AD patients^21^. We then measured the graph-theoretical metrics of representative functional segregation, including clustering coefficient and local efficiency, in the cortical areas above by applying graph theory on rs-fMRI analyses (36 elements = 2 metrics × 18 cortical areas in Fig. 2E), namely the TBSS-Graph method.

### The LMCI-related white matter alterations by MD-based TBSS

**Figure 3:**
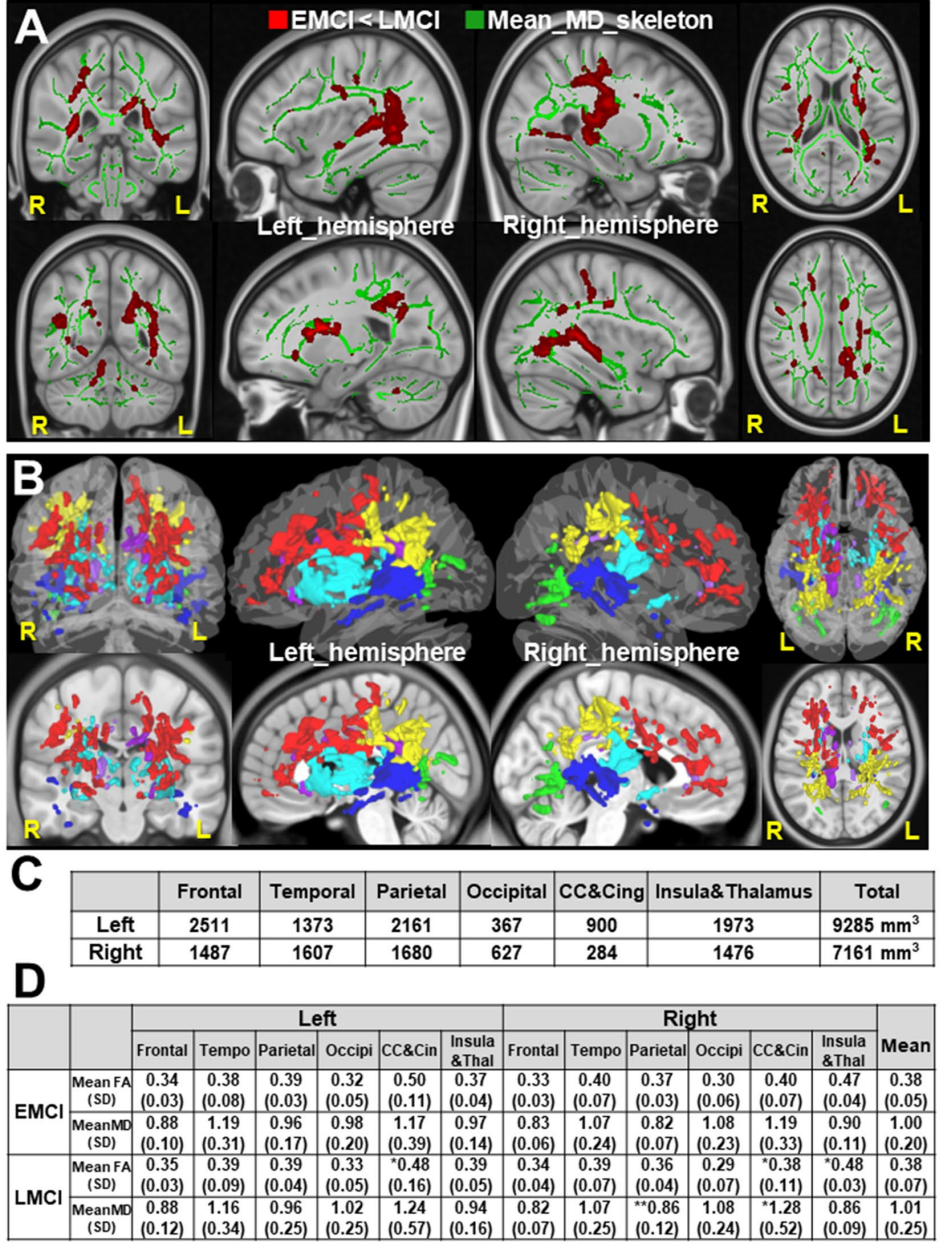
MD-based TBSS and Altered white matter regions. (**A**) The MD-based TBSS projects all subjects' MD data onto a mean MD tract skeleton before applying voxelwise cross-subject statistics (EMCI vs. LMCI). The registered average subjects’MD tract skeleton is represented in green, while LMCI-related white matter changes were represented in red color. The MD tract skeleton was overlaid on the coronal, sagittal, and axial T1-weighted MRI image (ICBM average brain). Left; coronal view, Middle; sagittal view of left and right hemisphere, Right; axial view. Significance level was *p* < 0.05 (Threshold Free Cluster Enhancement and Family-Wise Error corrected). (**B**) The LMCI-related white matter changes, identified by MD-based TBSS (EMCI vs. LMCI), were shown overlaid on the 3D glass average brain (upper images) and T1-weighted MRI image (lower images), respectively. The regions in the frontal, parietal, temporal, occipital lobe, Corpus Callosum (CC) and cingulum, and insula and thalamus regions were shown in red, yellow, blue, green, purple, and sky blue respectively. (**C**) The table shows the total volume (mm^3^) for LMCI-related white matter changes in each hemisphere by MD-based TBSS, which were sub-classed into frontal, temporal, parietal, occipital lobe, Corpus Callosum (CC) and cingulum (Cing), and insula and thalamus regions. (**D**) The table show mean diffusion parameters (mean FA and MD) in each ROI for LMCI-related white matter changes, which were sub-classed into frontal, temporal, parietal, occipital lobe, Corpus Callosum (CC) and cingulum (Cin), and insula and thalamus regions. *p < 0.1, **p < 0.05 (EMCI vs. LMCI, *t*-test). Statistical data are in Supplementary Fig. S3. *FA* fractional anisotropy, *MD* mean diffusivity, *TBSS* Tract-based spatial statistics, *CC* Corpus Callosum, *MCI* mild cognitive impairment, *EMCI* early MCI, *LMCI* late MCI.

We also conducted mean diffusivity (MD)-based TBSS to investigate LMCI-related WM alterations (Fig. 3A). The LMCI-related WM changes were shown in Fig. 3B, which were sub-classed into the frontal, parietal, temporal, occipital lobes, and Corpus Callosum (CC) and cingulum, and insula and thalamus regions. We measured the volume (mm^3^) of significant clusters of voxels by MD-based TBSS **(p** < 0.05**,** TFCE**-**corrected) in each region of bilateral LMCI-related WM changes (Fig. 3C). This result showed the left-hemispheric dominant LMCI-related WM alterations in volume (left; 9286, right; 7161 mm^3^ in total), especially in the frontal lobes (left; 2511, right; 1487), CC and cingulum (left; 900, right; 284). We also investigated the mean diffusion parameters (mean FA and MD) in each region of LMCI-related changes by MD-based TBSS (Fig. 3D). Although individual variation exists between each region, we found higher MD value in the right parietal lobe in LMCI subjects, compared with EMCI subjects. Statistical data of Fig. 3D is in Supplementary Fig.S3.

Then we prepared two additional feature subsets of diffusion parameters (FA, MD) in altered WM regions by gFA-based TBSS and MD-based TBSS (24 elements = 6 regions/hemisphere × 2 × (mean FA, MD)), respectively.

### Machine learning approach and performance for EMCI/LMCI classification

**Figure 4:**
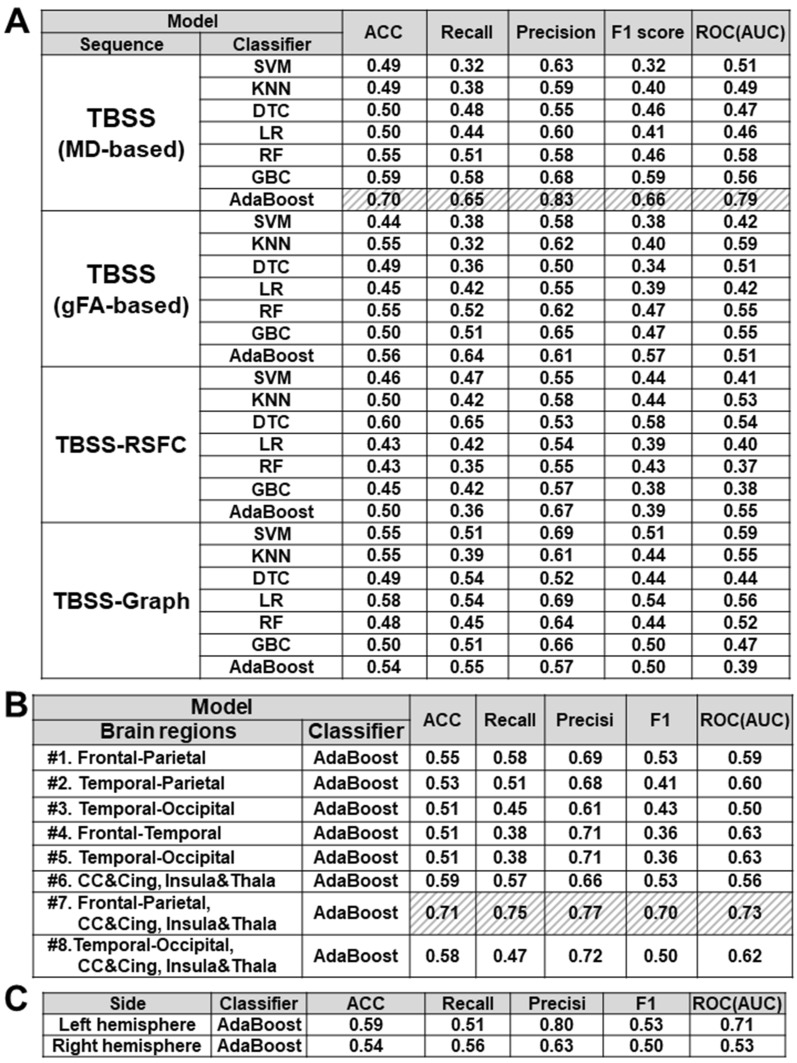
EMCI/LMCI classification performance in ML models with feature subsets. (**A**) The table indicates the EMCI/LMCI classification performance of ML models (SVM, KNN, DTC, LR, RF, GBC, AdaBoost), using four feature subsets by gFA-based TBSS, MD-based TBSS, TBSS-RSFC, and TBSS-Graph method. The performance was assessed by measuring mean accuracy (ACC), recall, mean precision, F1 score, and AUC (ROC). (**B**) The useful brain regions for EMCI/LMCI classification. With features extracted from each combination of brain regions, the classification performance of AdaBoost was evaluated by measuring mean accuracy (ACC), recall, mean precision, F1 score, and AUC (ROC). The brain regions were subclassified into each combination of #1. Frontal and Parietal lobe, #2. Temporal and Parietal lobe, #3. Temporal and Occipital lobe, #4. Frontal and Temporal lobe, #5. Temporal and Occipital lobe, #6.CC&Cing, Insula& Thalamus regions, #7. Frontal and Parietal lobe, Corpus Callosum (CC) and cingulum (Cing), and Insula and Thalamus regions. (**C**) The useful brain hemisphere for EMCI/LMCI classification. With features extracted from the right or left hemisphere, the classification performance of AdaBoost was evaluated by measuring mean accuracy (ACC), recall, mean precision, F1 score, and AUC (ROC). *RSFC* resting-state functional connectivity, *ROI* range of interest, *ML* Machine learning, *SVM* support vector machine, *KNN* k-nearest neighbor algorithm, *LR* Logistic Regression, *DTC* decision tree classifier, *RF* Random Forest, *GBC* gradient boosting classifier, *ACC* accuracy, *AUC* Area under the curve, *ROC* Receiver operating characteristic, *CC* Corpus Callosum.

In this study, the main purpose was to search for the optimal ML model for EMCI/LMCI classification. Using the four feature subsets above, we then adopted multiple ML classifiers to distinguish LMCI from EMCI subjects, including support vector machine (SVM), k-nearing neighbors (KNN), decision tree classifier (DTC), Logistic Regression (LR), Random Forest (RF), Gradient Boosting Classifier (GBC), and Adaptive Boosting Classifier (AdaBoost). We compared the classification performance of these multiple ML classifiers by calculating accuracy (ACC), Recall, Precision, F1 score, and area under the curve (AUC) of receiver operation curve (ROC), with tenfold cross-validation (CV). We took the mean of each metric to evaluate classification performance. The table showed AdaBoost classifier (in gray hatching of Fig. 4A), an ensemble ML algorithm, provides better performance of 70% accuracy and 79% AUC (of ROC), using features of diffusion parameters by MD-based TBSS.

We investigated which brain regions are useful for EMCI/LMCI classification. The altered WM areas by MD-based TBSS were sub-classed into six regions, including frontal, parietal, temporal, occipital lobes, Corpus Callosum (CC) and cingulum, and insula and thalamus. In Fig. 4B, then we addressed which combination of these regions provide better performance by AdaBoost classifier (i.e., #1 Frontal-Parietal lobe, #2 Temporal-Parietal lobe, #3 Temporal-Occipital lobe, #4 Frontal–Temporal lobe, #5 Temporal-Occipital lobe, #6 Corpus Callosum (CC)-Cingulum (Cing)-Insula-Thalamus, #7 Frontal-Parietal lobe-Insula-Thalamus, #8 Temporal-Occipital lobe-CC-Cing-Insula-Thalamus). The table in Fig. 4B showed the EMCI/LMCI classification performance of AdaBoost classifier using features from the combination of each region. The features from the #7 regions, including frontal, parietal lobes, CC and cingulum, and insula and thalamus, lead to better performance with 71% accuracy and 73% AUC (in gray hatching of Fig. 4B). In addition, features from the left hemisphere resulted in slightly higher performance with 71% AUC (Fig. 4C).

Finally, we compared our results to those in previous studies, which used ML classifiers for EMCI/LMCI classification (Table 2). Our result of 70% accuracy and 79% AUC by AdaBoost classifier was comparable to those in previous reports with 73–87% accuracy and 78–90% AUC^8,24–26,28,46,47^.

**Table 2:** Comparison with previous studies for EMCI/LMCI classification.

Authors	Target	Approach	Feature extraction	Feature selection	Classifier	ACC	AUC
Goryawala et al. (2015)	EMCI (114) vs. LMCI(91)	MRI (Cortical volume) + Neuropsychological scores	*SLRM for MRI and Neuropsychological test		*LDA	0.736	N/A
Jie et al. (2018)	EMCI (56) vs. LMCI(43)	DCN(dynamic connect. network) from rs-fMRI	Temporal & spatial Variability (DCNs)	*M2FL method	SVM	0.788	0.783
Nozadi et al. (2018)	EMCI(164) vs. LMCI(189)	FDG-PET, AV45-PET	Multimodal PET-MRI registration + ROIs-based or whole brain select		RF	0.725	0.79
Sheng et al. (2019)	EMCI(24) vs. LMCI(24)	BCT(Brain Connectivity Toolbox) from rs-fMRI	Network-based measures (BCT)	mRMR, Chi-square, Gini score,Kruskal–Wallis test, Fisher score(FS), Relief feature score	> 20Classifiers + *DNN	0.875 (SVM)	N/A
Zhang et al. (2019)	EMCI(33) vs. LMCI(29)	Graph theory (rs-fMRI)	3Network features + 3 freq. bands	mRMR, SS-LR, Fisher Score (FS)	SVM	0.838	0.905
Shi and Liu (2020)	EMCI(77) vs. LMCI(64)	rs-fMRI	Hilbert-Huang transform (HHT) Hilbert weighted frequencies(HWFs) Independent two-sample t-test		SVM	0.879	N/A

*SLRM stepwise linear regression models.

*M2FL method Manifold regularized multi- task feature selection.

*DCN dynamic connectivity network.

*LDA linear discriminant analysis.

*DNN deep neural network.

## Discussion

In the current study, we proposed several diffusion MRI-based ML approaches for EMCI/LMCI classification, based on the hypothesis that subtle brain changes could be detected earlier in white matter microstructures by diffusion MRI. Using four feature subsets extracted from single- or multi-modal MRI data including diffusion-MRI, we trained and tested multiple ML models and assessed performance with cross-validation. Our results indicated the single modal data of diffusion parameters (FA, MD) provide better performance than that of multi-modal method (TBSS-RSFC, TBSS-Graph method). The diffusion parameters of frontal, parietal lobe, Corpus Callosum, cingulum, insula, and thalamus were useful classification factors. In addition, those extracted from left hemisphere were slightly more useful for classification than right hemisphere. In general, different neuroimaging modalities could provide more essential complementary information than single modality^27^. However, our results showed the single modal features of diffusion MRI provided higher classification performance.

Our finding of left hemisphere dominant features for classification, which might reflect the more changed volumes of white matter in the left hemisphere, is compatible with a previous study. Goryawala et al. showed that significant features of brain volumes for EMCI/LMCI classification are from the left hemisphere^28^. These results suggest asymmetrical white matter alterations could occur during MCI progression. Additionally, our results of useful features from frontal, parietal lobe, and cingulum for classification are partially in agreement with previous studies. Hojjati et al. identified significantly different networks in MCI subtypes, including those in the frontal, temporal, and parietal gyrus^48^. Goryawala et al. showed the significant classification factors are cortical volumes of temporal, parietal, and cingulum for EMCI/LMCI classification^28^. Sheng et al., using graph theory metrics, selected features in the temporal or cingulate cortex^26^. Further, our findings suggest the association of insula and thalamus for classification of MCI subtypes. Numerous studies have revealed the insular gray matter loss^49^, dysfunction of insular network at the early stage of AD^50^, pre-symptomatic changes in thalamus^51^. These findings could reflect possible white matter alterations in the insula and thalamus during MCI progression.

Over the past decade, several ML approaches have been proposed for classification of AD and MCI. Current diagnostic methods for AD mainly depend on neuropsychological tests, neuroimaging, and biofluids, including cerebrospinal fluid (CSF) and serum^52^. Gurevich et al. and Kang et al. applied neuropsychological scores for discrimination of AD and cognitive impairment by ML^53,54^. Some studies used CSF and serum data for classification by ML^55,56^. A number of neuroimaging approaches have been applied in classification of AD and MCI, including positron emission tomography (PET) of Aβ-amyloid and tau deposition, structural MRI to detect brain atrophy, diffusion MRI and functional MRI^29,57–61^.

Although our results showed single modal features of diffusion MRI provided higher performance, a number of studies have effectively classified MCI subtypes by multi-modal MRI analyses with optimal feature selection^8,24–26,28,47,48^. In general, the feature matrix, which is extracted from MRI or PET analyses, contains a huge amount of irrelevant or redundant features. To remove irrelevant features and reduce feature dimensions, feature selection is typically performed before classification (Table 2). Goryawala et al. introduced a novel framework named SLRM (stepwise linear regression model) to combine MRI volumetric measures with neuropsychological scores^28^. Jie et al. compared the effect of feature selection methods between M2FL and gLASSO-based method, using the dynamic connectivity networks (DCNs)^47^. Nozadi et al. extracted ROIs as features by multimodal PET-MRI registration method^24^. Sheng et al. processed thousands of brain network features by filter and wrapper feature selection procedures^26^. Zhang et al. also used multiple brain network features and conducted feature selection by three different algorithms^8^. Shi and Liu extracted features by calculating the Hilbert weighted frequencies (HWFs) from decomposed rs-fMRI signals, with independent two-sample *t*-test as feature selection method for SVM^25^. Collectively, these results suggest the optimal feature selection from multi-modal MRI data might be critical to improve classification performance. Thus, previous studies have typically combined multi-modal features after extracting each single modal data, which is followed by optimal feature selection. In contrast, our method sequentially integrated the multimodality of diffusion MRI and rs-fMRI or graph theory. We presumed our sequential integration methods of multi-modalities (TBSS-RSFC and TBSS-Graph method) resulted in the over-reduction of features and lost the non-linear mutual relations. This might lead to the poor performance for classification.

In general, the white matter (WM) damage is considered to precede GM atrophy and network dysfunctions^29^. Our TBSS analyses showed LMCI-related white matter (WM) changes in the Corpus Callosum (CC). A number of studies using structural and diffusion MRI have revealed WM changes in the CC in neurological diseases, including AD, bipolar disorder, schizophrenia, and Huntington’s disease^62–66^. WM changes can develop as a consequence of a number of factors, including demyelination and decreased number of axons, and/or cortical grey matter (GM) atrophy^67,68^. It therefore remains unclear whether WM changes in the CC are specific to each disease. The two different mechanisms were proposed to cause CC atrophy in AD; the direct myelin damage of callosal fibers; and the cell death in the GM, particularly the large pyramidal cells in cortical layer III^69^. Assumingly, the WM changes in CC can affect inter-hemispherical communications. Vecchio et al. (2015) showed the FA reduction in CC by DTI analysis is associated with a loss of inter-hemispheric functional connectivity by resting-state EEG in MCI and AD patients^70^. Further, reduced FA and increased MD in cognition-related WM tracts (e.g. cingulum, superior longitudinal fasciculus) are correlated with MMSE score in AD patients^71^. These results suggest the WM alterations in MCI subjects could partly lead to the disrupted segregation of neural network in AD^21^. The pathophysiological process of AD reportedly starts 20 years or more before symptoms^2,3^. The deposition of Aβ-amyloid is one of early signs at preclinical AD stage. Several neuroimaging studies have shown the relationship between early white matter alterations and amyloid deposition with amyloid-β PET^72–75^. Although the results are not completely consistent, those studies have suggested white matter microstructural changes (reduced FA and increased MD values) can be correlated with Aβ-amyloid deposition. Taken together, these findings might support our hypothesis that subtle brain changes can be detected earlier by diffusion MRI data.

This study was subject to several limitations. Several issues need to be further addressed. First, the sample size of MCI subjects, especially that of LMCI, was limited in the ADNI-3 dataset**.** ADNI imaging was carried out at over than 50 imaging centers, using scanners from the three major MR vendors (GE, Siemens and Philips). Although ADNI MRI core has established a standard set of protocols and procedures (www.adni-info.org.), the different scanners could cause a potential inconsistency on the analyses of imaging data. Since ADNI-3 project is under progress, the clinical and neuropsychological information for each subject is limited. During preparing this paper, additional information of neuropsychological scores and biomarkers became available. We added the additional available information in Table 1 and Supplementary Figs. S1, S2. Based on recent studies, various neuropsychological scores and biomarkers could improve classification performance with neuroimaging studies, including MMSE, RAVLT, CSF protein levels, and Apolipoprotein-E (APOE) genotype^28,76^. The abnormal memory function in MCI was determined by a single memory score^2,4,5,7^, which could lead to misclassifications that cause low accuracy and specificity in the present study. Our results have to be verified with larger datasets and follow-up longitudinal studies to reduce individual variations and validate the proposed ML model.

In conclusion, the feature set of diffusion parameters in the regions of LMCI-related WM changes was useful to distinguish LMCI from EMCI subjects with application of ensemble ML algorithm.

## Supplementary Information

Supplementary Figure S1.

Supplementary Figure S2.

Supplementary Figure S3.

## Abbreviations

AD: Alzheimer’s disease
ADNI: Alzheimer’s disease neuroimaging initiative
EEG: Electroencephalogram
MCI: Mild cognitive impairment
EMCI: Early MCI
LMCI: Late MCI
MMSE: Mini-mental state examination
MRI: Magnetic resonance imagination
fMRI: Functional MRI
rs-fMRI: Resting-state fMRI
RSFC: Resting-state functional connectivity
SD: Standard deviation
CV: Cross-validation
ROI: Range of interest
ML: Machine learning
KNN: K-nearest neighbor algorithm
LR: Logistic Regression
DTC: Decision tree classifier
RF: Random Forest
SVM: Support vector machine
GBC: Gradient boosting classifier
AdaBoost: Adaptive Boosting
CC: Corpus Callosum
HCP: Human connectome project
HCP-MMP: Human Connectome Project multimodal parcellation
MNI: Montreal Neurological Institute
FA: Fractional anisotropy
gFA: Generalized FA
MD: Mean diffusivity
DMN: Default mode network
RSN: Resting-state network
ACC: Accuracy
ROC: Receiver operating characteristic
AUC: Area under the curve
WM: White matter
GM: Grey matter
