# Supplementary figures and images for: Searching for optimal machine learning model to classify mild cognitive impairment (MCI) subtypes using multimodal MRI data
Source: Sci Rep. 2022 Mar 11;12:4284. doi: 10.1038/s41598-022-08231-y (PMC8917197; doi:10.1038/s41598-022-08231-y)

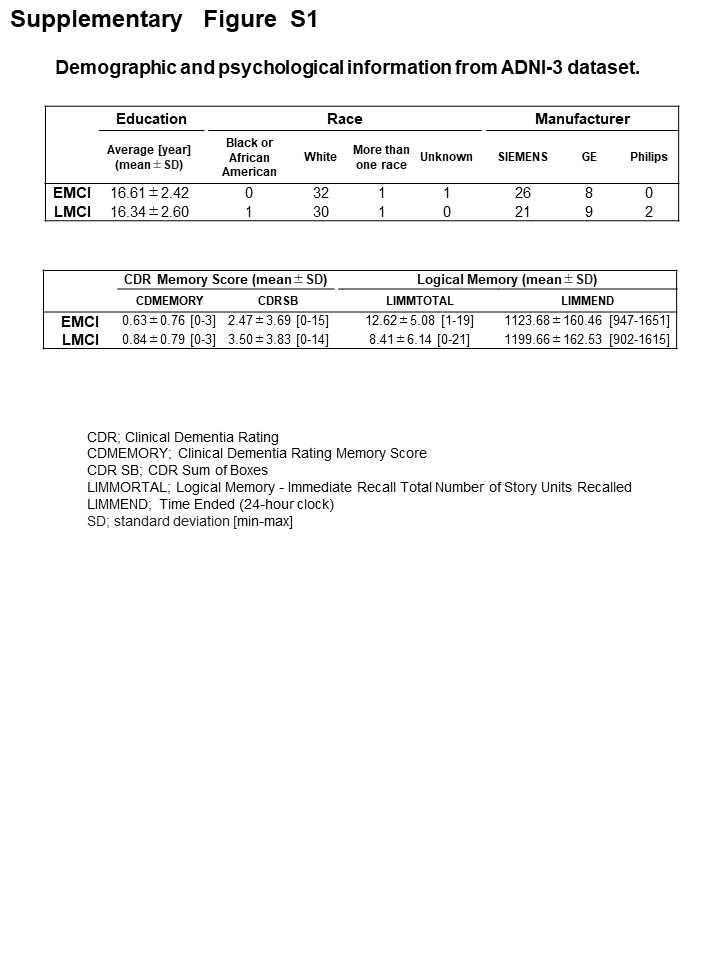

Supplement: Supplementary file 1 — Supplementary Figure S1. [file 41598_2022_8231_MOESM1_ESM.tif]

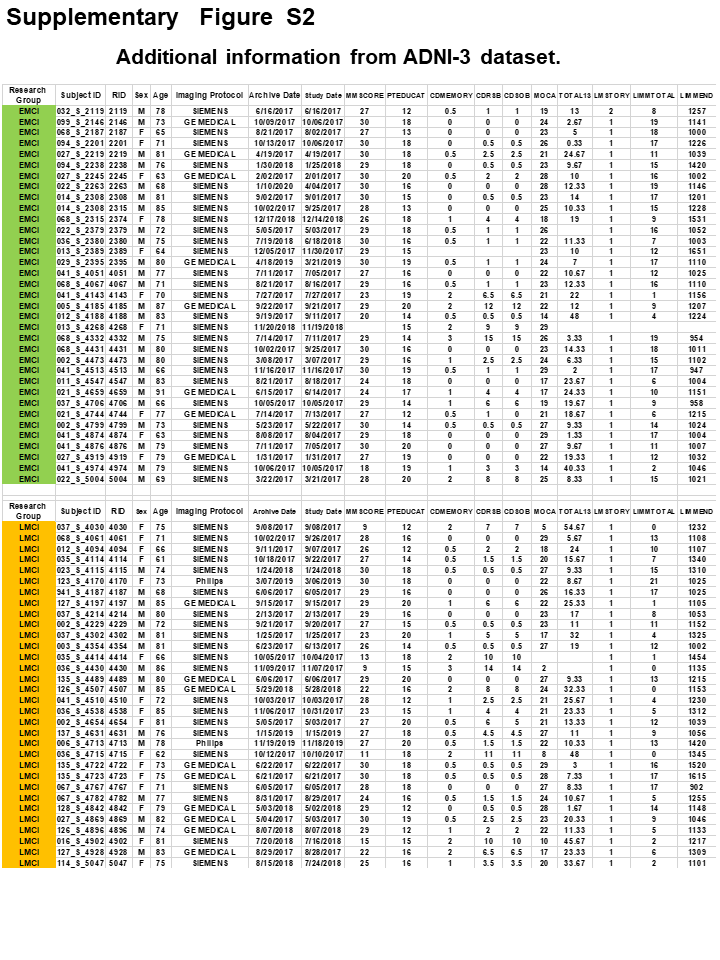

Supplement: Supplementary file 2 — Supplementary Figure S2. [file 41598_2022_8231_MOESM2_ESM.tif]

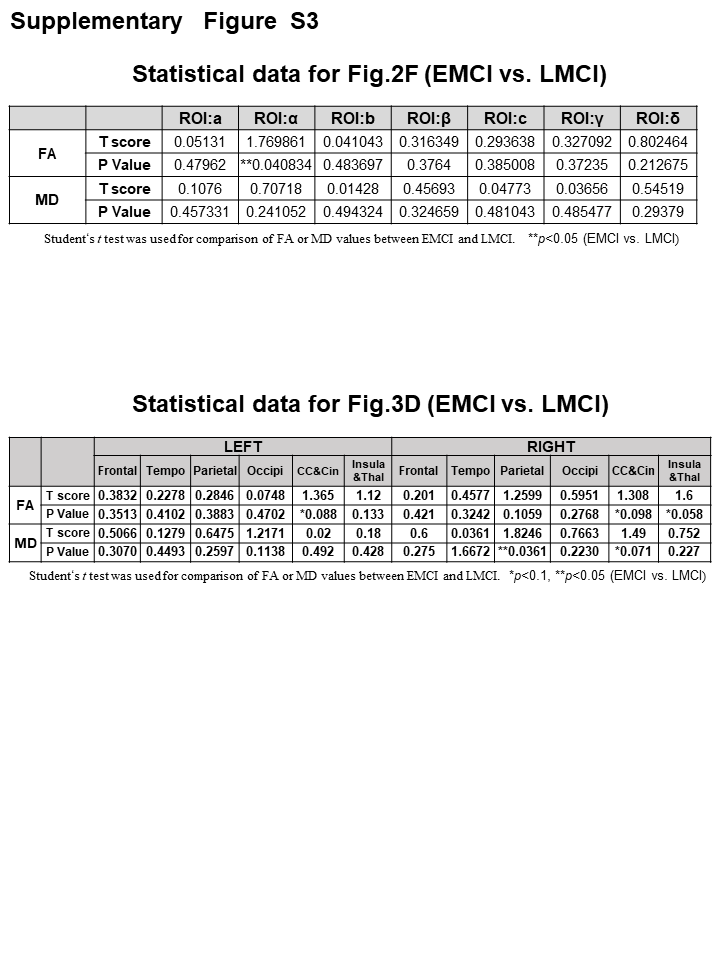

Supplement: Supplementary file 3 — Supplementary Figure S3. [file 41598_2022_8231_MOESM3_ESM.tif]
